# Massive fetomaternal hemorrhage: a case series and review of literature

**DOI:** 10.1515/crpm-2021-0079

**Published:** 2022-05-26

**Authors:** Carolina Smet, Luísa Queiró, Edmundo Santos, Ana Reis, Cristina Costa

**Affiliations:** Obstetricts and Gynecology Department, Hospital de São Francisco Xavier – Centro Hospitalar Lisboa Ocidental, Lisboa, Portugal; Neonatal Intensive Care Unit, Pediatrics Department, Hospital de São Francisco Xavier – Centro Hospitalar Lisboa Ocidental, Lisboa, Portugal; Clinical Pathology Department, Hospital de São Francisco Xavier – Centro Hospitalar Lisboa Ocidental, Lisboa, Portugal

**Keywords:** fetomaternal hemorrhage, neonatal anemia, sinusoidal cardiotocographic pattern

## Abstract

**Objectives:**

Massive fetomaternal hemorrhage (FMH) is a rare and difficult to diagnose event that can have catastrophic outcomes. Although many etiologies have been associated with FMH, the majority of cases are idiopathic and affect uncomplicated pregnancies. The prevailing symptom is decreased fetal movements but some cases are asymptomatic. Changes in the fetal Doppler ultrasound, a sinusoidal cardiotocographic pattern, neonatal anemia, unexplained hydrops or stillbirth can raise suspicion that such an event has occurred.

**Case presentation:**

This article presents a case series of severe FMH diagnosed in our center between 2011 and 2020 as well as a review of the current available literature.

**Conclusions:**

We highlight the importance of the clinician’s awareness on detecting this rare but potentially life-threatening event.

## Introduction

Fetomaternal hemorrhage (FMH) refers to the transfer of fetal blood into the maternal circulation that may occur at any stage of the pregnancy [1]. When in limited volume, it is considered a physiological event without clinical repercussion, whereas large volume FMH is associated with important morbidity and mortality [2].

The incidence of severe FMH is estimated to be around 0.3 to 1 in 1,000 births, depending on the volume of fetal blood loss considered meaningful [3].

Although many etiologies have been associated with FMH, the majority of cases have no identifiable cause. Pre-natal invasive diagnostic procedures, maternal trauma, external cephalic version and conditions leading to placental abnormalities (such as pre-eclampsia and chorioangiomas) are some of the situations that can contribute to FMH [1, 2, 4–6].

It is difficult to identify FMH cases since the majority present with unspecific symptoms and, depending upon its duration and severity, can display a broad spectrum of manifestations. It can be suspected in cases of unexplained stillbirth, hydrops, abnormal fetal heart rate tracings, maternal perception of decreased fetal movements or neonatal anemia [1, 2, 4–6].

When FMH is suspected, the middle cerebral artery peak systolic velocity (MCA-PSV) can help detect fetal anemia and some tests as the Kleihauer–Betke (KB) stain test, flow cytometry or high-performance liquid chromatography (HPLC) can be used to identify fetal hemoglobin in the mother’s circulation [4].

## Case presentation

We reviewed all the admissions in the neonatal intensive care unit (NICU) with the diagnosis of anemia due to massive FMH between 2011 and 2020. Massive FMH was defined as a severe fetal anemia without any other identified etiologies and a heightened percentage of fetal blood cells on the mother’s blood.

A total of 4 cases were identified in a period of 10 years which represents about 0.2 cases in 1,000 births.

**Figure 1:**
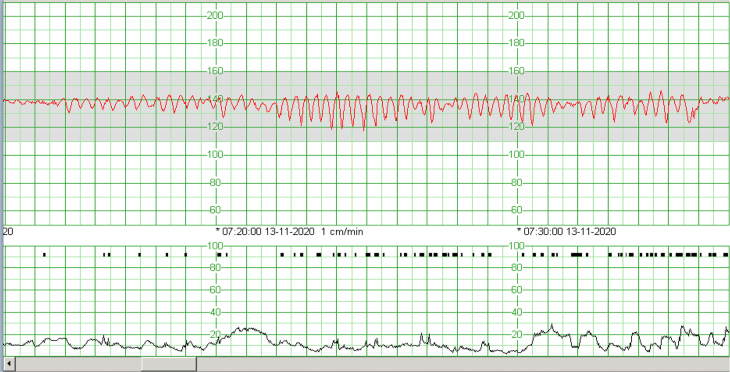
CTG of case 1 showing a sinusoidal pattern.

The collected data is displayed in Table 1. The age of the women ranges from 30 to 36 years, with a medium of 32 years. All of them were multiparous and had a Rhesus positive blood type. The average gestational age was 34 weeks+6 days with two term pregnancies, one late preterm and one early preterm. All the pregnancies had had a normal course and no risk factors for FMH were identified. Three cases presented with reduced fetal movements and one case was admitted for a non-reassuring cardiotocogram (CTG) with no symptoms. The CTG presented a sinusoidal pattern in one case, had late decelerations in two cases and reduced variability in three cases. The sinusoidal pattern detected in case 1 is shown below (Figure 1).

**Table 1:** Case reports.

	Case 1	Case 2	Case 3	Case 4
Age, years	34	30	32	36
Gravidity and parity	G2P1	G2P1	G4P1	G3P1
Blood type	0 Rh+	0 Rh+	A Rh+	A Rh+
Pregnancy course	Normal	Normal	Normal	Normal
Risk factors for FMH	None	None	None	None
Symptoms	Reduced FM	Reduced FM	Reduced FM	None
CTG	Sinusoidal pattern	Reduced variability	Reduced variability, late decelerations	Reduced variability, late decelerations
Gestational age (weeks+days)	38+1	29+0	35+1	37+3
Delivery	Cesarean	Cesarean	Cesarean	Cesarean
Sex	M	F	M	M
Weight, g	3,607	1,113	2,290	2,855
Percentile	50th–90th	10th–50th	10th–50th	10th–50th
Apgar score	2/4/5	0/3/4	3/7/7	8/8/9
Neonates’ Hb, g/dL	3.1	1.8	3.9	4.8
Fetal Hb in mother’s circulation, %	6.1	0.9	5.5	5.6
Direct Coombs test	Negative	Negative	Negative	Negative
Parvovirus B19 screening	NA	Negative	Negative	Negative
Treatment	Transfusion; advanced resuscitation	Transfusion; advanced resuscitation	Transfusion; advanced resuscitation	Transfusion
Outcome	Transferred to a hypothermia center	Neonatal death (5th day)	White matter lesions with neurological impairment	Discharged at 10 days of life, clinically well
Autopsy/placental findings	NA	No malformations. Intraventricular hemorrhage and fetal anemia compatible with FMH.	NA	NA

CTG, cardiotocogram; FM, fetal movements; M, male; F, female; Hb, hemoglobin; NA, non available.

In all cases the delivery was through urgent/emergent cesarean section. Three of the babies were male and one was female with weights ranging from 1,113 (10th–50th percentile) to 3,607 g (50th–90th percentile). The hemoglobin levels at birth ranged from 1.8 to 4.8 g/dL (normal levels at birth in newborns >34 weeks are 14 g/dL–22 g/dL; premature infants have slightly lower hemoglobin). In all cases, FMH was confirmed by a high-performance liquid chromatography of the mother’s blood showing increased fetal blood cells. The direct Coombs’ test was negative in neonatal circulation in all cases. Parvovirus B19 screening was performed in three cases, all with negative results.

At birth, all four newborns had visible pallor and needed transfusion of red blood cells concentrate. All but case 4 needed resuscitation measures that included invasive ventilation and volume resuscitation.

Regarding the treatment and outcome, case 1 (38 weeks+1 day of gestational age at delivery) was transferred to another hospital for therapeutic hypothermia. In addition to the hypoxic ischemic encephalopathy, there was also refractory hypoxemia associated with persistent pulmonary hypertension.

Case 2 (29 weeks+0 day at delivery) was admitted to the neonatal intensive care unit (NICU) in hypovolemic shock, with severe acidosis, progressing to severe intraventricular hemorrhage and eventually to neonatal death at the fifth day of life. The autopsy and placental analysis showed no signs of malformations in the neonate and confirmed the hypothesis of massive FMH.

Case 3 (35 weeks+1 day at delivery) was clinically stable from the second day of life. Cerebral ultrasound showed white matter alterations and the physical examination was unremarkable, with the exception of hyperreflexia and axial hypotonia. The cerebral lesions were ulteriorly confirmed by magnetic resonance imaging (MRI). The child is still currently followed by a neurodevelopment pediatrics team, maintaining (at 5 years of age) a neurological impairment in rehabilitation.

Case 4 (37 weeks+3 days) was transferred to the neonatal intensive care unit (NICU) with severe anemia. Except from pallor, there were no other signs or symptoms and, therefore, no need for other interventions besides a transfusion.

## Discussion

Massive FMH is a rare and difficult to diagnose event that can have catastrophic outcomes. Given its rarity, it’s difficult to have robust studies about this condition and the available evidence is based solely on case reports and case series.

It is estimated to affect around 0.3 to 1 in 1,000 births, representing the cause of 3.4% of all intrauterine deaths and 0.04% of all neonatal deaths [4, 7]. In our study, the estimated affected neonates were 0.2 in 1,000 births, less than the values presented in literature, which can be related with the fact that we included only live births, excluding the possible stillbirths due to this cause.

The threshold most frequently used to define a severe FMH ranges between 30 and 150 mL, but some studies advocate that the definition should regard the percentage of total fetal-placenta blood volume, which varies with the gestational age and fetal weight [3]. However, the time frame of the hemorrhage, as well as the fetal mechanisms available to counteract it, are more relevant to assess a severe FMH rather than the magnitude of the blood loss. For example, a hemorrhage of 100 mL that occurs throughout a week can be well tolerated by the fetus, whereas the same amount of blood lost on the course of hours can cause cardiovascular distress, culminating in a stillbirth [3, 5].

The gestational age of the fetus might also play an important role in its response to the blood loss. In our series, the neonate with the worst outcome (Case 2) was an early preterm (29 weeks+0 day) with probably fewer mechanisms to compensate the bleeding. Even though it was the case with the smallest percentage of fetal cells in the mother’s circulation (0.9%), the loss of that quantity for a neonate with 1,113 g of weight was very likely to be significant in absolute terms.

There are many etiologies that have been associated with FMH, but about 80% of the cases has no apparent explanation [3]. Described causes are related to a placental barrier disruption such as maternal trauma, external cephalic version or pre-natal invasive diagnostic procedures. All our cases were low-risk uneventful pregnancies and no precipitating factor was identified.

Recognition of FMH may only become apparent after the injury to the fetus has already occurred. The most frequently described symptom is the mother’s perception of decreased or absent fetal movements, which is a nonspecific symptom. Therefore, many cases are only detected after birth in anemic neonates. In our series, three cases presented with decreased fetal movements and one case was asymptomatic and admitted for a pathological CTG in a routine check-up.

Fetal anemia can have multiple etiologies, that can be divided into three groups: hemorrhagic anemia (which is the most frequent), hemolytic anemia and hypoplastic anemia. The hemorrhagic causes include anomalies of the umbilical cord or placenta, FMH, obstetric trauma and defects in hemostasis. In the hemolytic anemia group, the most common cause is isoimmunization caused by Rh, AB0 or minor groups incompatibility. It is also important to consider sepsis, TORCH congenital infections and congenital erythrocyte defects. Anemia caused by low erythrocyte production is less common, and usually presents itself after 48 h of life [8]. In our series, there was no report of placental or umbilical anomalies that could justify the severe anemia observed in all the newborns. All women had a Rhesus positive blood type and all the newborns had a negative direct Coombs test, which excludes severe isoimmunization disease as a possible cause. Parvovirus B19 infection was tested in 3 cases and was negative. No other infectious or congenital etiologies were identified.

A sinusoidal fetal heart rate pattern in the CTG has often been associated with fetal anemia, but this pattern is not pathognomonic: it can also be present in cases of hypoxia and fetal acidosis, after administration of analgesics (e.g. Pethidine) or in cases of amnionitis [6]. Moreover, in previous studies this pattern seems to be much less common than other abnormal tracings identified in FMH, such as reduced or absent variability, decelerations or bradycardia [4,5,6]. In our series, all cases presented a pathological CTG, (one of them with a sinusoidal pattern), leading to an urgent/emergent cesarean.

When FMH is suspected, some tests can be used to identify fetal hemoglobin in the mother’s circulation. The Kleihauer–Betke (KB) stain test is the most frequently used test and relies on the fact that hemoglobin F (Hb F) is resistant to acid elution whereas adult hemoglobin is not. To perform this test, a peripheral blood smear is incubated in an acid solution and then stained, resulting in a dark-pink coloration of the cells who contain mostly fetal hemoglobin compared with the maternal erythrocytes which appear uncolored. The fetal cells are then counted and reported as a percentage of the adult cells. This test requires a manual count leading to interobserver and interhospital variations. Besides, the fetal cells have a lifespan of approximately 100 days before being eliminated in the maternal serum at an unpredictable rate, which can be accelerated in cases of ABO or Rh incompatibility. Also, the Hb F content in fetal cells decreases with advancing gestational age which may lead to poor staining if the hemorrhage occurs near term [3, 9].

Flow cytometry is considered the gold standard test for FMH diagnosis because it is an automated and more accurate test, but it is more expensive and not available in every hospital [9].

When none of these tests is available, like it happened in our center, high-performance liquid chromatography (HPLC) can be used. It relies on the differences between the various hemoglobin fractions, on the absorption by a stationary chromatography column, and on the subsequent elution by a mobile phase, that constitutes the liquid that circulates through the column. Hemoglobin fractions are identified by their retention time and quantified, in percentage, by the area corresponding to the elution profile peak. This test is inexpensive, simple, reliable and it is usually more available than flow cytometry. This test does not allow to quantify the amount of blood transferred in the FMH and, just as the KB test, it does not distinguish fetal cells from adult F-cells [10].

Neither one of these three tests answers the crucial question of when did the fetal cells entered the maternal circulation [3].

When a fetal anemia is suspected, the increase in the MCA-PSV might help to confirm it. Fetal anemia causes an increase in the cardiac output and a preferential distribution of the oxygen to the brain causing an increase in blood velocity in the cerebral arteries. This tool has an estimated false-positive rate of 12% so it is advisable to reserve it for cases with a strong suspicion [3].

If severe FMH is detected during a pregnancy with a viable fetus, the obstetrician is left with two options: either deliver or maintain the pregnancy. The last option will oblige to a cordocentesis with intrauterine blood transfusion and steroids for lung maturation if indicated. This decision is extremely challenging and must weigh the risk of prematurity vs. the complications of cordocentesis and the uncertainty of the posterior evolution of the hemorrhage. The gestational age and the availability of a physician skilled in cordocentesis must also be taken into account [2, 3]. If the pregnancy is near-term, emergency delivery and blood transfusion of the neonate is the advised option [2].

In our center, obstetricians skilled in cordocentesis are not available, which explains the decision to deliver the early premature with 29 weeks gestational age.

The short and long-term neonatal prognosis are difficult to predict and depend on several variables such as the volume of blood loss in relation with the total fetal blood volume, the velocity of the bleeding and the fetal compensatory mechanisms. Untreated anemia can result in cardiac failure, hypovolemic shock, neurologic injury, persistent pulmonary hypertension or neonatal death [3]. Given the limited scientific data, the real incidence of these outcomes remains uncertain.

## Conclusions

Severe FMH is a fortunately rare but severe complication, that generally affects uneventful pregnancies in the third trimester. The true incidence of significant FMH probably remains underreported due to an heterogenous clinical presentation and insufficient recognition among physicians. Antenatal diagnosis warrants a heightened index of suspicion as the presenting signs are usually nonspecific. This diagnosis should be considered in cases of persistent maternal perception of decrease fetal movements, and an abnormal CTG should prompt further diagnostic tools, such as MCA-PSV or the quantification of fetal hemoglobin in the maternal circulation. A timely identification of these cases is essential to improve the neonatal outcome by performing an immediate resuscitation after delivery and having a blood supply ready for a rapid transfusion.
